# Expert Elicitation on Exposure to Tick Bites and Tick-Borne Encephalitis Risk in Occupational and Recreational Forest Activities

**DOI:** 10.3390/v18010082

**Published:** 2026-01-08

**Authors:** Claude Saegerman, Elsa Quillery, Marc Leandri, Véronique Raimond, Pauline Kooh, Philippe Fravalo, Thierry Hoch, Yves Hansman, Nathalie Boulanger

**Affiliations:** 1 Fundamental and Applied Research for Animal and Health (FARAH) Center, University of Liege, 4000 Liege, Belgium; 2 Risk Assessment Department, French Agency for Food, Environmental and Occupational Health and Safety, 94700 Maisons-Alfort, France; 3 UMI SOURCE, UVSQ-Université Paris-Saclay, IRD, 78286 Guyancourt, France; 4 Social Science, Economy and Society Department, French Agency for Food, Environmental and Occupational Health and Safety, 94700 Maisons-Alfort, France; 5 USC Metabiot, Le Cnam, 22440 Ploufragan, France; 6 Oniris, INRAE, BIOEPAR, 44300 Nantes, France; 7 Service de Maladies Infectieuses et Tropicales, CHU de Strasbourg, 1 Place de L’hôpital, UR3073—PHAVI—Pathogen-Host-Arthropod Vector Interactions University of Strasbourg, 67000 Strasbourg, France; 8 UR3073—PHAVI—Pathogen-Host-Arthropod Vector Interactions—Ticks and Tick-Borne Disease Group, France and French Reference Centre on Lyme Borreliosis, CHRU, University of Strasbourg, 67000 Strasbourg, France

**Keywords:** tick, tick borne encephalitis, forest activities Europe, experts, elicitation, tick-bite exposure, awareness

## Abstract

**Background:** Tick-borne encephalitis (TBE) virus is transmitted to humans via tick bites and occasionally via the consumption of unpasteurized milk products. According to the literature, the most important driver of TBE emergence and increase in incidence in humans is changes in human behaviour/activities. **Method and principal findings:** To compensate for the lack of data, expert opinions were gathered to identify the risk factors for exposure to tick bites linked to twenty-eight human activities (professional or recreational) in forests and to target prevention messages at the populations most at risk. Opinions were elicited from a total of twenty-five European experts. Seven criteria were included in the analysis for each activity: frequency, seasonality, duration of exposure, distance covered, degree of contact with vegetation, speed and average level of protection against tick bites. The activities considered to be the most at risk of exposure to tick bites are, in descending order: three occupational activities (forest monitoring activities, forestry and wood industry activities and scientific and/or analytical activities), five recreational activities and one hunting activity (mushroom picking, spending the night in the forest, hunting, naturalist activities, orienteering, and berry or fruit picking). **Conclusions and significance:** Prevention messages regarding tick bites could be targeted at people who engage in activities considered in this analysis to be at highest risk of exposure to tick bites.

## 1. Introduction

Prevention against tick-borne diseases is an important public health issue. Lyme disease due to Borrelia is the most frequent worldwide tick-borne disease infection. However, the tick-borne encephalitis virus (TBEV) responsible for tick-borne encephalitis (TBE) is identified as a potential emerging pathogen in Western Europe. This virus is transmitted to humans by the bites of infected *Ixodes* ticks and, in rare cases, via the consumption of unpasteurized milk products [1]. The economic impact of the TBE in Europe is significant but highly variable, depending on the study [2,3,4,5]. TBE is endemic in parts of Asia and Europe, and according to the literature, the most important driver of TBE emergence and increase in incidence in humans is changes in human behaviour/activities [6]. For example, increasing leisure time spent outdoor activities and increasing travel and mobility within Europe are two drivers of TBE risk [7].

Forests are ideal locations for recreational activities (leisure, relaxation, tourism, discovering flora and fauna, hunting) as well as professional activities (professional activities in forest environments, including both forestry operations and knowledge-oriented work). Human forest visitation in Europe is trending upward, driven by a growing demand for recreation, well-being, and tourism, a trend significantly amplified by the COVID-19 pandemic [8,9]. In fact, COVID-19-related lockdowns significantly reduced the incidence of various infectious diseases by decreasing social contact. However, the incidence of TBE was not affected due to the pursuit of forest activities [10].

The risk of exposure to tick bites depends on several factors that favour tick-human encounters, such as certain leisure or professional activities [11,12]. For example, at the microhabitat level, exposure depends on the areas frequented by humans and the manner in which they are managed. More precisely, in forested areas, walking on maintained paths results in fewer tick bites than walking on grassy trails or off-trails. Vegetation maintenance therefore plays a role in exposure to tick bites [13]. Other exposure factors involve the relationship between seasons, weather conditions and human behaviour. For example, it has been hypothesised that tick bites are more frequent on weekends with good weather following a week of precipitation, which is a favourable time for mushroom picking [14]. Exposure to tick bites also depends on the density of *Ixodes ricinus* questing on vegetation and is therefore higher in mainland regions in spring and early summer, which also corresponds to the period of greatest human activity in forest areas.

In this context, identifying the activities that pose the greatest risk is a critical task in the development of awareness and information campaigns targeting people who practice these activities to reduce their exposure to tick bites. This paper aims to rank forest activities (professional or recreational) according to a series of criteria and European expert opinion.

## 2. Materials and Methods

### 2.1. Context and Expert-Opinion Elicitation

The following context was considered in the expert-opinion elicitation process: a forest in which all factors are present to favour the presence of ticks (presence of hosts, biotic and abiotic factors) and TBEV circulation. In this forest, all of the activities listed are possible, with consideration of the most frequently encountered situation for each type of activity (assignment of a score per criterion).

A questionnaire was prepared in the form of an Excel file consisting of various sheets:

▪ Sheet entitled ‘Expert information’: this was intended to briefly describe the profile of the expert completing the questionnaire.
▪ A set of seven sheets concerning the various criteria considered: (1) frequency of the activity; (2) seasonality; (3) duration of exposure; (4) distance covered; (5) degree of contact with vegetation; (6) speed; and (7) average level of protection against tick bites. These sheets were designed to gather the opinion of each expert by assigning a score for each of the criteria defined for each activity. Each sheet in the file provided the necessary instructions and details (e.g., definitions of activities and scores) (Table 1 and Table 2).
▪ Sheet entitled ‘weighting of criteria’: this was intended to distribute a fixed number of points (*n* = 70) among the seven various criteria mentioned above. If the expert considered the criteria to be equivalent, he/she assigned the same number of points to each. Otherwise, he/she assigned the number of points in proportion to the relative importance of the criteria considered.
▪ Sheet entitled ‘uncertainty’: for each criterion, they were also asked to give an uncertainty rating between 1 (minimum uncertainty) and 5 (maximum uncertainty).

Each tab in the Excel file explained the approach and details required to complete the task. The questionnaire was refined considering comments following a pre-test of the questionnaire with four experts from the Anses working group, each with a different area of expertise (political science, economics, ticks and tick-borne diseases, risk assessment). The questionnaire was then sent to a list of scientific experts compiled by the task force.

### 2.2. Scoring System and Clustering of Activities

Considering, on the one hand, the score assigned for each criterion and the number of possible options for each score and, on the other hand, the relative weight of each criterion, a weighted overall score (WOS) was calculated for each activity and for each expert using the following formula:

(1) WOS = ∑ (Score C*i*/M C*i*) × Weight C*i*

where C*i* is a given criterion and M is the number of modalities per score for a given criterion (Table 2). As the number of modalities is not the same for each criterion, each score awarded was divided by the number of modalities for the criterion in question (standardization requirement).

The activities were then ranked in relation to each other based on the medians of the overall scores obtained for each activity by all experts. Finally, the activities were aggregated (clustered) into four groups using a regression tree analysis (activities classified as high risk, at risk, moderately at risk and low risk). Each group of activities was identified by a given average and by minimizing its standard deviation as much as possible.

### 2.3. Sensitivity Analysis

In order to ascertain whether the ranking of forest activities was influenced by the selection of experts, a sensitivity analysis was performed using a comparison of ten bootstraps each (random selection of fifteen experts amongst twenty-five) with the ranking of forest activities with all experts elicited as reference. The difference between the above ranking of forest activities (each bootstrap versus all experts as reference) was tested using the Pearson coefficient of correlation test [6]. Because of the overrepresentation of French experts in the European sample, a sensitivity analysis was also performed, excluding these experts. If this coefficient was close to 1 and the *p*-value was less than 0.05, the correlation between the two rankings of forest activities tested was considered significant.

### 2.4. Uncertainty Analysis and Additional Statistical Analysis

For each criterion, uncertainty was estimated using a rating between 1 (minimum uncertainty) and 5 (maximum uncertainty). A violin plot was used to depict the weight and the uncertainty about the seven criteria considered. It is a statistical visualization that combines a classical box plot (vertical axis) with a kernel density plot (horizontal axis) to show the distribution of numeric data across one or more groups [15].

## 3. Results

### 3.1. Number of Experts and Their Field of Expertise

**Figure 1:**
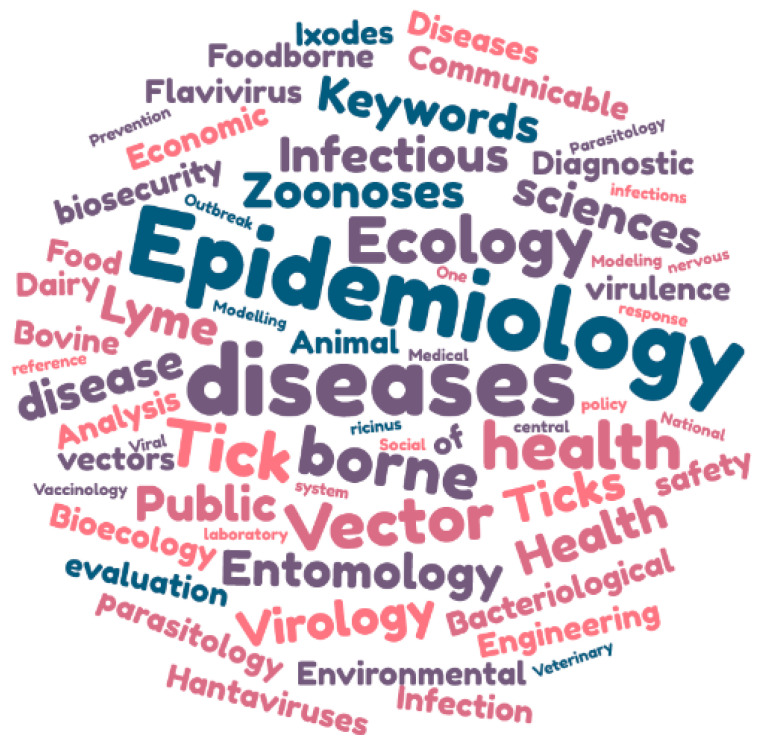
Keyword cloud characterising the expertise of the twenty-five experts consulted.

Opinions were elicited from a total of twenty-five European experts. Their recruitment was done by the French Agency for Food, Environmental and Occupational Health and Safety using a list of experts in vectors and vectorial-borne diseases. These experts came from the following countries (in decreasing order): France (*n* = 14); Belgium (*n* = 3); Slovenia (*n* = 2); Sweden (*n* = 2); and Italy, Lithuania, the United Kingdom, and Switzerland (*n* = 1 each). The keywords characterising the expertise of these experts are depicted in Figure 1 and corroborate the multidisciplinary nature of the study. The top three keywords were epidemiology (13 occurrences), tick-borne diseases (7 occurrences), and vector-borne diseases (4 occurrences). The number of years of professional expertise in the field of tick-borne diseases followed a normal distribution (Shapiro–Wilk test, *p*-value = 0.68) with a mean of 21.1 years and a standard deviation of 9.1 years.

The panel of experts consisted of one research engineer, two with master’s degrees in science, twelve with PhDs, and ten others with PhDs who were professors. Four of them worked for an (inter)national agency, eight for a laboratory or research institution, two for a Ministry of Health, and eleven for a university.

### 3.2. Relative Importance of Criteria and Their Uncertainty

**Figure 2:**
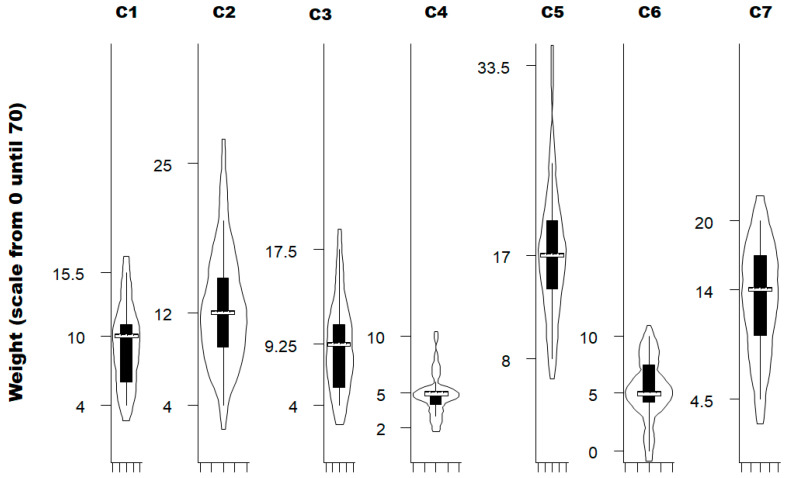
Violin plot with the weighting rating for each criterion estimated by the experts consulted (*n* = 25). C1, Frequency of activity; C2, Seasonality of the activity; C3, Duration of exposure for one activity session; C4, Distance covered during the activity; C5, Contact with vegetation during the activity; C6, Speed during the activity; C7, General protection against ticks biting. The white bold horizontal line represents the median of the weight attributed for each criterion by experts; the extremities of each rectangle represent, respectively, the first and third quartiles; adjacent lines to the whiskers represent the limits of the 95% confidence interval.

Not all criteria carry the same weight (Kruskal–Wallis test for equality of populations; Chi2 (6 degrees of freedom; α = 0.05) = 95.14; *p*-value = 0.0001) (Figure 2). Using quantile regression, the results show that the degree of contact with vegetation (criterion 5; *p*-value < 0.001) and the level of general protection against tick bites (criterion 7; *p*-value = 0.001) are more important than activity frequency, seasonality, and duration of exposure (criteria 1 to 3, respectively), while distance covered during activity (criterion 4) and speed during activity (criterion 6) are significantly less important (*p*-value < 0.001).

**Figure 3:**
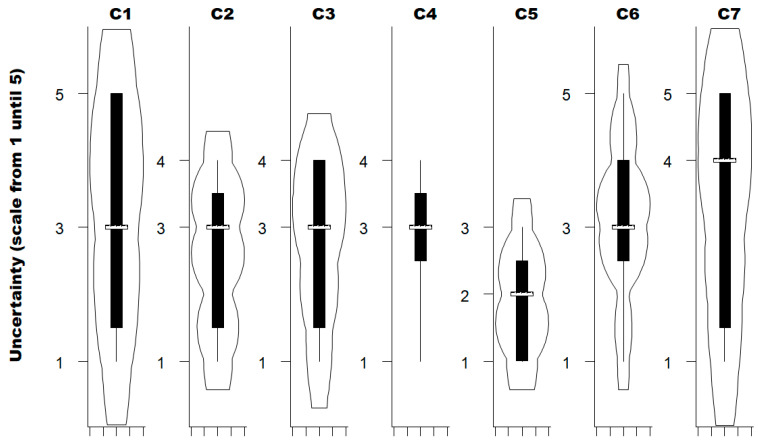
Violin plots with the uncertainty rating for each criterion estimated by the experts consulted (*n* = 25). C1, Frequency of activity; C2, Seasonality of the activity; C3, Duration of exposure for one activity session; C4, Distance covered during the activity; C5, Contact with vegetation during the activity; C6, Speed during the activity; C7, General protection against ticks biting. The white bold horizontal line represents the median of the level of uncertainty attributed by experts using a scale from 1 (minimal uncertainty) to 5 (maximum uncertainty); the extremities of each rectangle represent, respectively, the first and third quartiles; adjacent lines to the whiskers represent the limits of the 95% confidence interval.

The uncertainty regarding the criteria to be considered when prioritising forestry activities was assessed by each expert on a scale ranging from 1 (minimal uncertainty) to 5 (maximum uncertainty) (Figure 3). The median uncertainty rating was 3 for the frequency of activity (C1), its seasonality (C2), the duration of exposure for one activity session (C3), the distance covered during the activity, and the speed during the activity (C6). For the contact with vegetation during the activity (C5) and the general protection against tick bites (C7), the median uncertainty was 2 and 4, respectively. No statistical difference in the median uncertainty was observed between criteria (Kruskal–Wallis test for equality of populations; Chi2 (6 degrees of freedom; α = 0.05) = 8.05; *p*-value = 0.23).

### 3.3. Ranking and Clustering of Forest Activities

**Figure 4:**
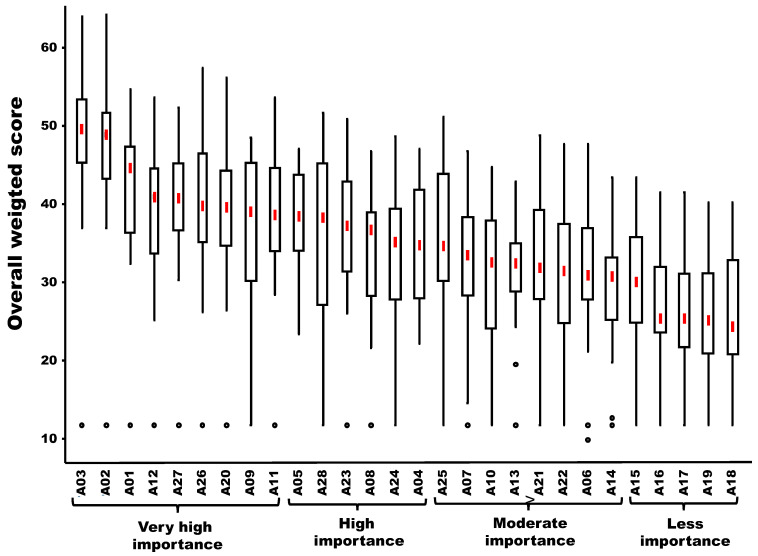
Box plot showing the ranking of forestry activities in descending order of risk of exposure to tick bites (the descending order is based on the median values of the overall weighted score obtained by the twenty-five experts). A01 to A28 are the different activities listed in Table 1. The red vertical line represents the median; the solid lines at the top and bottom of each rectangle represent, respectively, the first and the third quartiles; adjacent lines to the whiskers represent the limits of the 95% confidence interval; small circles represent outside values.

The ranking and clustering (in four different groups) of forest activities in descending order of risk of exposure to tick bites is presented in Figure 4. The first group of activities with very high importance included nine forest activities (in decreasing order): forest protection activities (A03), forestry and wood industry activities (A02), scientific and/or analytical activities (A01), mushroom picking (A12), spending the night in the forest (A27), hunting as shooting and battue (A26), naturalist activities (A20), orienteering (A09), berry or fruit picking (A11). In addition, three other groups (high importance, moderate importance and less importance) were identified and included six, eight and five activities, respectively.

### 3.4. Sensitivity Analysis

The result of the sensitivity analysis indicated that, irrespective of the experts excluded, excluding some experts only had very limited or no significant effects on the ranking of forest activities compared to the reference (all experts elicited). Indeed, using ten bootstraps of fifteen experts amongst twenty-five, the Pearson correlation coefficient between each bootstrap against the ranking of twenty-five experts as a reference was very high (value between 0.97 and 0.99, with a *p*-value < 0.0001). The normality of *p*-value was also verified (Shapiro–Wilk test; *p*-value = 0.37) with a mean of 0.98 and a standard error of 0.007. Because of the overrepresentation of French experts in the European sample, a sensitivity analysis was also performed, excluding these experts, and limited effect on the ranking of forest activities was observed (value of the Pearson correlation coefficient 0.97 with a *p*-value < 0.0001).

## 4. Discussion

To the best of the author’s knowledge, no study was published before concerning the typology of forest activities based on weighted criteria related to the risk of human exposure to tick bites. The present study contributes to filling this gap.

Amongst twenty-eight forest activities included in this study, the first group of nine activities was more at risk of tick bites (in decreasing order): firstly three occupational activities (forest protection activities, forestry and wood industry activities and scientific and/or analytical activities) and secondly, five recreational activities and one hunting activity (mushroom picking, spending the night in the forest, hunting by shooting and beating, naturalist activities, orienteering, and berry or fruit picking).

This expert-opinion elicitation involved twenty-five experts with different fields of expertise and disciplines. It is legitimate to wonder whether the size of the expert panel is sufficient and whether its composition is appropriate to produce reliable results from the elicitation process. Currently, there is no standard method to calculate the required size of a panel of experts. However, ref. [16] summarized some trends. Indeed, a recommendation for panel size is five to twenty experts with diverse knowledge [17]. Moreover, under typical circumstances, the panel is usually between 10 and 30 experts [18,19,20,21,22,23,24,25,26,27,28,29]. However, the extent of expertise of the panel (partially and indirectly tested in the study using the sensitivity analysis) is far more important for decision-making effectiveness than the number of participants [30,31]. Finally, it is highly unlikely that another equally expert group will produce radically different results from a panel of fifteen experts [32]. Based on evidence (publications in international peer-reviewed journals), both the composition and the size of the expert panel (*n* = 25) involved in the present study are adequate and in accordance with the best practices in the field of expert-opinion elicitation. In addition, our sensitivity analysis demonstrates the robustness of the proposed expert-opinion elicitation, for which no important bias was observed.

Considering the seven criteria included in the analysis, one of them concerns the risk of tick bites associated with forest activities (i.e., the degree of contact with vegetation), and another concerns protective measures (i.e., the level of general protection against tick bites). The degree of contact with vegetation like sitting on grass during work or taking a break during an activity is most often associated with highest frequency of tick bites in the literature [33]. In general, prevention against tick bites relies mainly on mechanical and chemical prevention [34]. Preventive measures include wearing light-coloured clothing that covers the skin (long sleeves, long trousers) so that ticks can be easily detected and using skin repellents [35]. Suitable products include DEET (N,N-diethyl-3-methylbenzamide), IR3535 (synthetic ethyl butylacetylaminopropionate), picaridin, and oil of lemon eucalyptus or PMD (para-menthane-diol) [36]. Depending on the product used, some restrictions exist in case of pregnancy, breastfeeding, and for children [37]. Other preventive measures include regular checks of clothing and skin for ticks and careful removal of the ticks if found (if possible, with a tick remover or fine-tipped tweezers if the tick is already attached), and visits limitation to areas where ticks are abundant during the seasons when they are most active, especially by staying on paths. Despite the existence of marketing authorisation for clothing impregnated with pyrethroids, particularly permethrin, the risk-benefit balance of their use is now considered unfavourable for use by the general population and is therefore no longer recommended [38]. Prevention policies and information campaigns can improve awareness of the risk of tick bites and associated diseases, as well as the adoption of effective protective measures. Several studies support this view, particularly those relating to Lyme disease. For example, in this regard, between 2016 and 2019 in France, the rate of use of skin repellents and long clothing in situations of exposure increased by 2 and 7 percentage points, respectively, reaching 18% and 73% [39]. Given that the Lyme plan launched in France in 2016 aimed to raise awareness of these risks, it can be assumed that part of this change is due to information campaigns. Indeed, awareness of Lyme disease, which is better known than TBEV, remained at only 41% in 2019, compared with 29% in 2016 [39]. In the case of Lyme disease, a positive correlation was identified in France between protecting oneself and feeling well-informed [39]. This correlation has also been proven in the United Kingdom [40] and the Netherlands [41]. Information and prevention policies relating to protection against tick bites, such as those implemented in the Lyme plan since 2016 in France [34], are among the relevant courses of action recommended to limit human infections with TBEV. However, some of the protective measures generally recommended against Lyme disease are not effective against TBEV. Indeed, ‘ex-post’ measures such as body inspection or corrective measures such as the use of a tick remover to remove ticks as quickly as possible would not prevent TBEV transmission, which occurs immediately after the bite [42,43], unlike infection with the bacterium responsible for Lyme disease, which occurs later [44]. In this regard, continuing the information campaigns initiated in the Lyme plan can help to further increase individual protection, with an emphasis on those that also work for TBEV. More broadly, the implementation of prevention and information options regarding tick bites can build on existing tick-related initiatives like annual awareness day organised by the reference centres for tick-borne diseases or awareness-raising through citizen surveillance network on tick biting (e.g., CiTIQUE in France, TickNet in Belgium, TekenRadar in The Netherlands or the App Tick Prevention in Switzerland). Continued education must be tailored to the areas and target audiences (e.g., endemic versus emerging regions, children versus adults, urban areas versus forest areas). It can also take the form of dedicated signage, such as information boards in high-risk areas or messages in specific applications, to warn of the presence of ticks. Raising public awareness could also be achieved through better training for community pharmacists [45].

## 5. Conclusions

According to a systematic review and meta-analysis, it is important to note first that outdoor activities provide health benefits and promote well-being [46]. To compensate for the lack of data, expert-opinion elicitation was undertaken to identify the risk factors for exposure to tick bites linked to twenty-eight human activities in forests and to target prevention messages at the populations most at risk. Prevention messages regarding tick bites could be targeted at people who engage in activities considered in this analysis to be at the highest risk of exposure to ticks (in particular, mitigation measures should be presented in the following operational sequence for each user: what should I do before, during, and after each activity?). More observational studies are needed to gain more evidence-based information on tick bites related to forest activities, with the aim of improving risk assessment modelling. The implementation of a similar study is recommended in other continents, especially Asia where TBEV is more endemic and virulent [47], and for a broader number of forest activities.

## Figures and Tables

**Table 1:** List of twenty-eight forestry activities that were considered in the expert elicitation.

Activity	Definition
*(* *i) Professional activities*	
A01. Scientific and/or analytical activities	Activities related to experimentation, prospecting, or collection in forests by naturalists, entomologists, geologists, botanists, mycologists, etc.
A02. Forestry and timber industry activities	Forestry activities aimed at maintaining forests for commercial exploitation (reforestation, planting, forest regeneration) or forestry operations, covering activities related to timber harvesting in the broad sense (felling, skidding, etc.).
A03. Forest protection activities	Activities related to the protection or monitoring of a specific forest area. The professionals involved are mainly forest rangers, forest naturalists, etc.
*(ii) Leisure activities*	
A04. Walk	A walk is a pedestrian route undertaken for the purpose of going outdoors to relax or exercise.
A05. Hiking	Hiking is an outdoor sporting activity that involves planning and following a route on foot, without running.
A06. Nordic walking	Nordic walking is a dynamic form of walking in the countryside. It combines endurance and aerobic exercise with fast walking using special walking poles.
A07. Jogging in a wooded area (slow running)	Running for physical exercise.
A08. Trail	A trail run is a running event that takes place in a natural environment.
A09. Orienteering	It is a race against the clock on varied terrain, on a course marked with checkpoints that competitors must find by following routes of their choice, using a map and compass.
A10. Fitness trail	This is a sporting walk punctuated by a series of physical or meditative activities, usually in a natural setting.
A11. Picking berries or fruit	An activity that involves picking edible fruits (especially berries), seeds, leaves, stems, or roots from certain plants found in nature.
A12. Mushroom picking	An activity that involves picking mushrooms in nature.
A13. Mountain bike	Mountain biking is a cycling activity practised on rough terrain, away from paved roads.
A14. Touring bike	Touring cycling is a sporting and leisure activity that involves riding on paths and trails at varying speeds.
A15. Horse riding	Horse riding, which can be mounted or harnessed, is an outdoor leisure activity. It is also referred to as outdoor riding or nature riding.
A16. Off-road motorcycling	Motorcycling activities that take place on dirt roads or unpaved tracks that are more or less wild. This includes enduro, trail and touring activities.
A17. Quad	An activity that involves driving an open-top, single-seater or two-seater, four-wheeled off-road vehicle for trail riding.
A18. Tree climbing	An outdoor sport practised in forests, combining climbing and moving from tree to tree along a secure course.
A19. Climbing	A sport that involves climbing a rock face to reach the summit of a mountain.
A20. Nature activities	Naturalists’ activities include observing, protecting, photographing and analysing ‘natural beings’ in the fields of botany, zoology and mineralogy.
A21. Forest therapy (forest bathing), relaxation	A practice that involves connecting with nature to improve mental and physical health.
A22. Fishing (pond and river)	Fishing is the activity of catching aquatic animals (fish, crustaceans, cephalopods, etc.) in their forest habitat (streams, ponds, lakes, pools).
A23. Lying/sitting on the grass	An activity that involves staying in a stationary position on the ground, in the grass, to rest, contemplate nature or eat (picnic).
A24. Outdoor games	Recreational activities that take place outdoors in an unstructured and freely chosen manner. These may include sports games (pétanque, frisbee, ball games, etc.), children’s games, treasure hunts, life-size role-playing games, etc.
A25. Wood gathering/harvesting (cutting wood for private individuals)	In several countries, cutting or collecting wood in forests is strictly regulated. Wood gathering is the right of local residents to cut wood on communal land.
A26. Spending the night in the forest	This may involve spending the night in the forest in a more or less basic camp, usually as part of an activity such as scouting, camping or bivouacking for a hike.
*C. Hunting activities*	
A27. Hunting as shooting and battue	Hunting with firearms (shotguns, rifles or bows), which can be practised individually or in groups, without dogs or with one or more dogs.
A28. Stalking	Hunting from a hide, in which the hunter waits for game in a specific location, hidden and ready to shoot.

**Table 2:** Criteria to be considered when prioritising forestry activities and the different scores to be assigned for each criterion.

Criterion (Number of Score Modalities)	Score	Definition
Frequency of activity (4 modalities)	1	Very occasionally (a few times a year)
	2	Occasionally (once or twice a month)
	3	Fairly frequently (once or twice a week)
	4	Frequently (more than twice a week)
Seasonality (4 modalities)	1	Never during the tick season
	2	Sometimes during the tick season
	3	Frequently during the tick season
	4	Still during the tick activity period
Duration of exposure (4 modalities)	1	Less than 1 h
	2	Between 1 and 4 h
	3	Between 4 and 8 h
	4	More than 8 h
Distance covered during the activity (5 modalities)	0	Less than 1 km
	1	Between 1 and 5 km
	2	Between 5 and 20 km
	3	Between 20 and 50 km
	4	More than 50 km
Degree of contact with vegetation (5 modalities)	0	Negligible to none
	1	Low
	2	Medium
	3	High
	4	Very high
Speed during the activity (5 modalities)	0	On-site activity
	1	Less than 3 km/h
	2	Between 3 and 8 km/h
	3	Between 8 and 13 km/h
	4	More than 13 km/h
Overall level of protection against tick bites (9 modalities)	0	No protection
	1	Non-specific protection provided by clothing or protection related to the activity performed (e.g., motorcycle suit).
	2	Specific use of protective clothing
	3	Specific use of skin repellents
	4	Body inspection after exposure
	5	Use of two specific means of protection: wearing protective clothing and using skin repellents
	6	Use of two specific means of protection: wearing protective clothing and body inspection after exposure
	7	Use of two specific means of protection: use of skin repellents and body inspection after exposure.
	8	Use of three specific means of protection: wearing protective clothing, use of skin repellents and body inspection after exposure

## Data Availability

Data is contained within this article.
